# mRNA Levels of Imprinted Genes in Bovine *In Vivo* Oocytes, Embryos and Cross Species Comparisons with Humans, Mice and Pigs

**DOI:** 10.1038/srep17898

**Published:** 2015-12-07

**Authors:** Zongliang Jiang, Hong Dong, Xinbao Zheng, Sadie L. Marjani, David M. Donovan, Jingbo Chen, Xiuchun (Cindy) Tian

**Affiliations:** 1 Center for Regenerative Biology, Department of Animal Science, University of Connecticut, Storrs, Connecticut, 06269, USA; 2 Institute of Animal Science, Xinjiang Academy of Animal Science, Urumqi, Xinjiang, P.R. China; 3 Department of Biology, Central Connecticut State University, New Britain, Connecticut, 06050, USA; 4 Animal Biosciences and Biotechnology Laboratory, Agricultural Research Services, United States Department of Agriculture, Beltsville, Maryland, 20705, USA

## Abstract

Twenty-six imprinted genes were quantified in bovine *in vivo* produced oocytes and embryos using RNA-seq. Eighteen were detectable and their transcriptional patterns were: largely decreased (*MEST* and *PLAGL1)*; first decreased and then increased (*CDKN1C* and *IGF2R)*; peaked at a specific stage (*PHLDA2*, *SGCE, PEG10, PEG3, GNAS, MEG3, DGAT1*, *ASCL2*, *NNAT*, and *NAP1L5)*; or constantly low (*DIRAS3*, *IGF2*, *H19* and *RTL1)*. These patterns reflect mRNAs that are primarily degraded, important at a specific stage, or only required at low quantities. The mRNAs for several genes were surprisingly abundant. For instance, transcripts for the maternally imprinted *MEST* and *PLAGL1,* were high in oocytes and could only be expressed from the maternal allele suggesting that their genomic imprints were not yet established/recognized. Although the mRNAs detected here were likely biallelically transcribed before the establishment of imprinted expression, the levels of mRNA during these critical stages of development have important functional consequences. Lastly, we compared these genes to their counterparts in mice, humans and pigs. Apart from previously known differences in the imprinting status, the mRNA levels were different among these four species. The data presented here provide a solid reference for expression profiles of imprinted genes in embryos produced using assisted reproductive biotechnologies.

Genomic imprinting involves a series of precisely regulated epigenetic processes that cause genes to be expressed in a parental-origin-specific manner in mammals^1^. Proper allelic expression of imprinted genes is important in embryonic and placental development as well as in maternal behavior^2^. Apart from their unique expression pattern, the level of imprinted gene expression is also a pivotal part of genomic imprinting. The exact numbers of total imprinted genes and their roles in mammalian development remain open questions. It is estimated that there are approximately 150 and 100 imprinted genes in mice and humans, respectively (http://igc.otago.ac.nz/Search.html and http://www.mousebook.org/imprinting-gene-list), (for original references, see http://www.mousebook.org/catalog.php?catalog=imprinting). The identification of imprinted genes in livestock species, however, lags behind^3^, with a total of 28, 17, and 10 confirmed, imprinted genes in cattle^4^, pigs^5^, and sheep^6^,^7^,^8^, respectively (http://www.geneimprint.com/site/genes-by-species.Ovis+aries).

The specific genes imprinted in each species can also be very different. For example, only 50 imprinted genes in the mouse overlap with those in humans, and numerous genes imprinted in the mouse and/or humans are not imprinted in the other species or in farm animals^9^. Moreover, the timing of imprinting activation during development is also species- and developmental stage-specific. Monoallelic expression is seen in mouse embryos as early as the two-cell stage and is observed for most imprinted genes by the blastocyst stage^10^. However, the allelic expression status of most imprinted genes is not known in human embryos^11^. To date, the onset of imprinted expression of imprinted genes in livestock species has not been examined systematically, but is known to occur much later than the blastocyst stage in bovine, ovine and porcine embryos^7^,^12^,^13^,^14^,^15^,^16^.

The exact nature of genomic imprints is still relatively unknown. It is known that genomic imprinting is regulated through epigenetic mechanisms, specifically allele-specific DNA methylation at differentially methylated regions established during gametogenesis and embryogenesis^17^, that are maintained in subsequent cell divisions during pre-implantation development^18^. Global DNA methylation patterns in zygotes and early embryos from several species have been studied and found to differ dramatically. For example, the male pronucleus is nearly completely demethylated in the mouse and rat, partially demethylated in cattle and goats, and minimally demethylated in sheep and pigs^19^,^20^. In addition to DNA methylation, imprinting regulation likely involves other epigenetic mechanisms, such as histone modifications, chromatin architecture, and non-coding RNAs^9^,^18^.

Imprints are established during gametogenesis and imprint maintenance can be disrupted during embryo development by environmental factors, such as *in vitro* culture and associated manipulations^21^. Increased incidences of imprinting disorders, including large offspring syndrome (LOS) in ruminants and Beckwith-Wiedemann Syndrome (BWS) in humans, have been reported in assisted reproductive technologies (ART) where *in vitro* culture of oocytes/embryos is routine^22^,^23^. Because differences in the transcriptomes between *in vitro* and *in vivo* embryos have been identified^24^,^25^, quantitative analysis of imprinted gene expression profiles in *in vivo* pre-implantation embryos can serve as the essential gold standard to which embryos produced from various biotechnologies can be compared.

To date, the expression of only a selected few imprinted genes have been characterized and shown to be regulated in a tissue- and/or developmental stage-specific manner across species, including humans^26^, porcine^5^,^15^,^27^,^28^,^29^ and bovine^12^,^30^,^31^. The recently available comprehensive RNA sequencing (RNA-seq) profiles of pre-implantation pig^32^, cattle^24^,^33^, mouse^34^ and human^34^,^35^ embryos, led us to use these robust datasets to analyze the abundance of the mRNAs of 26 bovine genes whose imprinting status has been previously confirmed. These genes were also compared across the above-mentioned four species during oocyte and embryo development. Our data provide important evidence for stage- and species-specific differences of imprinting during pre-implantation development and will serve as an important reference for embryos produced using assisted reproductive biotechnologies.

## Results

The total number of imprinted genes in the bovine genome is still unknown. From the 28 confirmed imprinted genes in the bovine, *MAOA* and *XIST* were excluded from our analysis because they are only imprinted in trophectoderm-derived cells^36^,^37^. The mRNA abundance of imprinted genes in MII oocytes and embryos were compared within and amongst four different species: cattle, humans, mice and pigs. Overall, 18 of the 26 confirmed bovine imprinted genes were detected in bovine *in vivo* oocytes and/or pre-implantation embryos (Supplementary Table S1), while only 14, 12 and 9 of these were expressed in human, mice and pig embryos, respectively. Among them, the levels of six genes, cyclin-dependent kinase inhibitor 1C *(CDKN1C),* GNAS complex locus *(GNAS),* insulin-like growth factor 2 receptor *(IGF2R),* mesoderm specific transcript *(MEST),* pleckstrin homology-like domain, family A, member 2 *(PHLDA2)* and pleiomorphic adenoma gene-like 1 *(PLAGL1),* were high (RPKM >10) in bovine oocytes or in at least one of the bovine embryonic stages (Supplementary Table S1), while others were expressed at relatively low levels. The mRNAs detected here are likely do not reflect imprinted expression since such an expression pattern is established relatively late in the bovine^38^.

The changes of the 18 expressed imprinted genes were categorized into five different dynamic patterns according to their abundance during pre-implantation development: continuously decreasing, decreased then increased, peaked at a specific embryonic stage, remained low until the morula/blastocyst stage, or constantly lowly expressed from oocytes to the blastocyst stage. These patterns reflect mRNAs that are primarily degraded, important at a specific stage, such as the maternal-zygotic transition or morula or blastocyst stage, or only required at low quantities.

**Figure 1:**
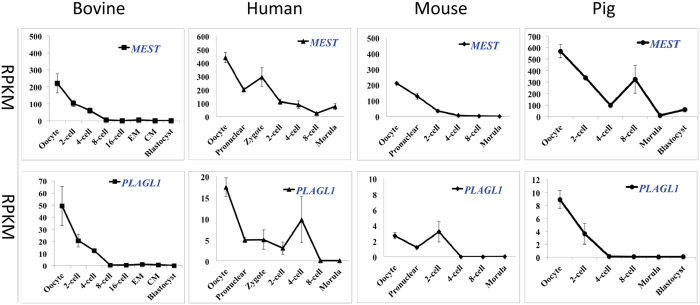
Levels of transcriptional expression of bovine imprinted genes that continuously decreased during pre-implantation development (mean ± SEM). Paternally expressed genes are labeled in blue.

The first group, which included the maternally imprinted *MEST* (also known as the paternally expressed gene 1 (*PEG1*)) and *PLAGL1*, represents genes that had an overall trend of decreasing abundance during pre-implantation development in the bovine. The same trend was also seen in the other three species (Fig. 1). Specifically, *MEST* and *PLAGL1* were highly expressed in oocytes; the expression was dramatically decreased from MII oocytes to the 8-cell stage and then was maintained at a low but detectable level up to the blastocyst stage. While having an overall similar trend, the transition to low levels occurred at different stages in the other species. Specifically, we observed earlier decreases at the 2- or 4-cell stage in the mouse and pig embryos; while, the change in humans was similar to that observed in cattle (i.e., decreased at the 8-cell stage). Notably, the *MEST* mRNA level in the oocytes was the highest among all genes studied in all four species. *PLAGL1* was also seen at relatively high levels in oocytes from all species examined. Among all the genes studied, these two genes were also consistent in their imprinting status (maternally imprinted) in the four species. Due to the late onset of monoallelic expression of imprinted genes in the bovine, it is likely that the mRNAs detected here are transcribed from both parental alleles. Nevertheless, since both *MEST* and *PLAGL1* are maternally imprinted, it is reasonable to expect that the maternal alleles of these genes in the oocytes carry expression-inhibitory imprints established during gametogenesis. However, their high expression levels in the oocytes indicate that the genomic imprints on the maternal alleles of these genes are either not established or not recognized at this stage of development.

**Figure 2:**
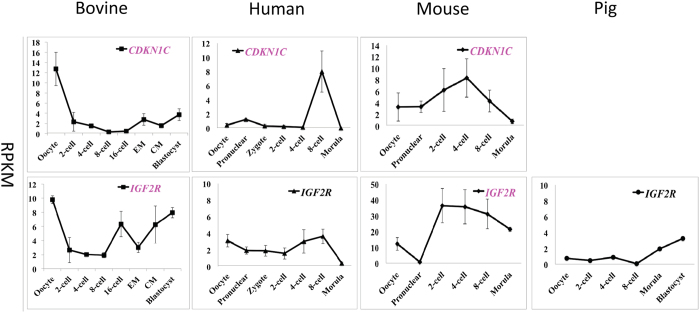
Transcriptional expression of bovine imprinted genes that were decreased first and then increased during bovine pre-implantation development (mean + SEM). Maternally expressed genes are labeled in pink and genes that are not imprinted in a particular species are labeled in black. The lack of a graph indicates that the gene was not detected in that species.

The second dynamic expression pattern was displayed by the paternally imprinted *CDKN1C* and *IGF2R* and represents genes with expression that first decreased and then increased during pre-implantation development (Fig. 2). It is likely that this pattern is accounted for by initial mRNA degradation followed by active transcription when the gene products are needed for embryonic development. Interestingly, the mRNA dynamics of these two genes were different in the other three species studied. In human, mouse and pig oocytes only low levels of *CDKN1C* and *IGF2R* were found. The levels then increased and then decreased in the human and mouse embryos, but continued to increase in the pig *(IGF2R).* Furthermore, *CDKN1C* was not detectable in pig oocytes or embryos (Fig. 2).

**Figure 3:**
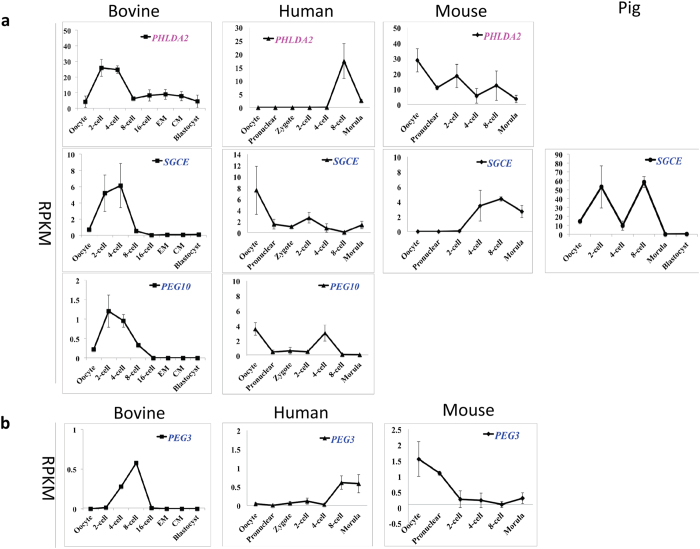
Transcriptional expression of bovine imprinted genes that increased first and then decreased at the 2- or 4-cell stage (**a**), or at the 8-cell stage (**a**) (mean ± SEM). Maternally and paternally expressed genes are labeled in pink and blue, respectively. Genes that are not imprinted in a particular species are labeled in black. The lack of a graph indicates that the gene was not detected in that species.

The third group represents genes in the bovine embryos whose mRNA levels peaked at a specific embryonic stage and subsequently maintained a relatively constant level to the blastocyst stage (Fig. 3a,b). For example, the paternally imprinted *PHLDA2,* the maternally imprinted sarcoglycan epsilon (*SGCE*) and the paternally expressed gene 10 (*PEG10*) all peaked at the 2- to 4-cell stages, while the paternally expressed gene 3 (*PEG3*) peaked at the 8-cell stage. Interestingly, members of this group were not all expressed in the other species. For instance *PHLDA2*, *PEG10*, and *PEG3* were not detectable in pigs, and *PEG10* was not expressed in mice. This observation and the drastically different expression patterns shown in Fig. 3 suggest significant species variations in genetic imprinting during early embryo development.

**Figure 4:**
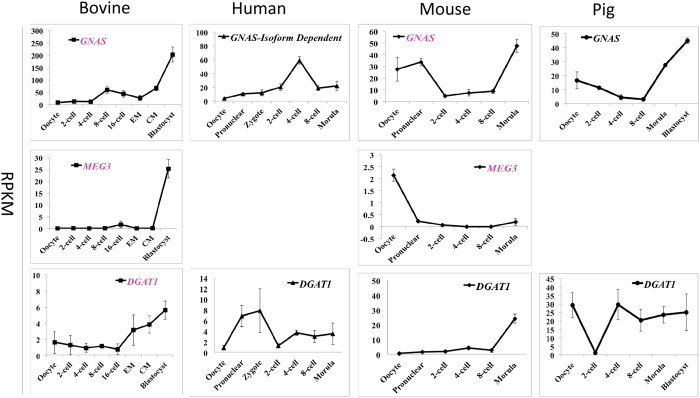
Transcriptional expression of bovine imprinted genes that maintained relatively low expression and then peaked at blastocysts to high levels (mean ± SEM). Maternally and paternally expressed genes are labeled in pink and blue, respectively. Genes that are not imprinted in a particular species are labeled in black. The lack of a graph indicates that the gene was not detected in that species.

**Figure 5:**
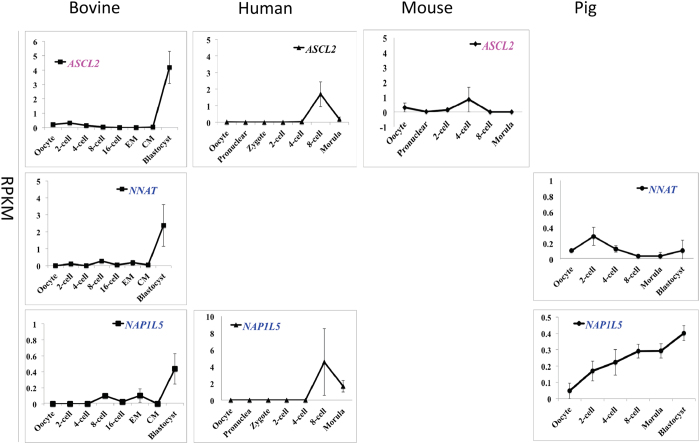
Transcriptional expression of bovine imprinted genes that maintained relatively low expression and then peaked at blastocysts to low levels (mean ± SEM). Maternally and paternally expressed genes are labeled in pink and blue, respectively. Genes that are not imprinted in a particular species are labeled in black. The lack of a graph indicates that the gene was not detected in that species.

The fourth group included the paternally imprinted *GNAS*, maternally expressed gene 3 (*MEG3*), diacylglycerol O-acyltransferase 1 (*DGAT1*), achaete-scute family bHLH transcription factor 2 (*ASCL2*), and the maternally imprinted neuronatin (*NNAT*) as well as nucleosome assembly protein 1-like 5 (*NAP1L5*). These genes maintained relatively low expression levels only peaking at the morula or blastocyst stage in the bovine (Figs 4 and 5). Major species differences were also seen in the dynamics of these genes. For example, *DGAT1* peaked at the zygotic stage in humans and 4-cell stage in pigs. *MEG3* and *GNAS* accumulated in the mature mouse oocytes, yet were barely detectable in the bovine oocytes. This group also contained the most inconsistencies in imprinting status among the four species. For example, *GNAS* was not imprinted in either humans or pigs and *DGAT1* is only imprinted in cattle.

**Figure 6:**
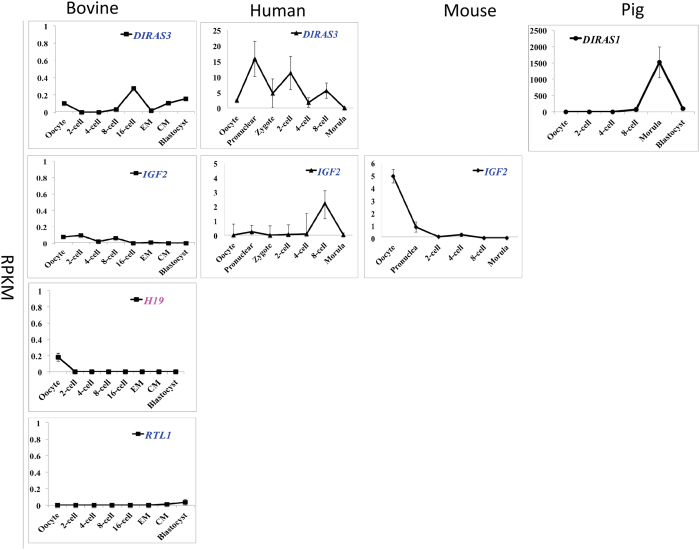
Transcriptional expression of imprinted genes that maintained low expression during pre-implantation development (mean ± SEM). Maternally and paternally expressed genes are labeled in pink and blue, respectively. Genes that are not imprinted in a particular species are labeled in black. The lack of a graph indicates that the gene was not detected in that species.

The last group was comprised of the maternally imprinted DIRAS family, GTP-binding RAS-like 3 (*DIRAS3*), insulin-like growth factor 2 (*IGF2*) and retrotransposon-like 1 (*RTL1*), as well as the paternally imprinted and non-coding RNA, *H19*. These genes maintained relatively constant low levels of expression throughout all stages studied in bovine oocytes and embryos (Fig. 6). Interestingly, these genes were not as silent in the other species. For example, *DIRAS3* was expressed at an extremely high level in pig morulae and was relatively high in multiple human embryonic stages. High levels of the maternally imprinted *IGF2* was observed in the mouse oocytes yet completely absent in pigs. Of note, neither *H19* nor *RTL1* were detected in human, mouse and pig oocytes or embryos. The near undetectable levels of *H19* in all bovine embryonic stages and the failure to detect the transcript in the oocytes/embryos of other species is consistent with low expression in bovine ovary^39^.

In addition to these categories based on the bovine gene expression patterns, we identified eight genes that are imprinted in the bovine and transcribed in other species, but were not detectable in bovine oocytes/embryos. Three genes, including small nuclear ribonucleoprotein polypeptide N (*SNRPN*), tumor suppressing subtransferable candidate 4 (*TSSC4*), ubiquitin specific peptidase 29 (*USP29*) were expressed in the human embryos, and four, including *SNRPN*, *TSSC4, USP29*, antisense transcript gene of *PEG3* (*APEG3*), were expressed in the mouse embryos (Table 1 and Supplementary Table S2).

It is important to point out that using the RNA-seq data, we were able to directly compare levels of gene expression between embryo stages and species. For example, the mRNA levels for *MEST* and *PLAGL1* were the highest compared to the barely detectable, *H19* and *IGF2*. This information is not available from previous studies employing real time PCR, where the mRNA levels of selected imprinted genes were expressed as percentages of control mRNA set at 100%^12^,^14^,^19^,^38^,^40^.

To confirm the bovine RNA-seq results, we performed quantitative real-time PCR (qRT-PCR) on five genes using bovine *in vivo* oocytes, 4-cell and blastocyst stage embryos (n = 3). The selected genes represented gene expression patterns in the following categories: largely decreased (*MEST* and *PLAGL1)*; first decreased and then increased (*CDKN1C*); peaked at a specific stage (*PHLDA2*); and low until the blastocyst stage (*GNAS*). The qRT-PCR detected greater fold changes in most cases and substantiated results from RNA-seq (Table 2).

## Discussion

Imprinted genes play critical roles in normal fetal and placental development. Interestingly, gene imprinting is not only developmental stage-specific, but also species-specific. Mammalian genomic imprinting has primarily been studied in mice and humans, while only limited information is available in livestock species. Due to species variations, most information gained from mouse and human studies cannot be extended to other species. In this study, we provide the first comprehensive description of total transcript levels of currently known and confirmed bovine imprinted genes during bovine *in vivo* embryonic development and in three other mammalian species. We showed that the expression profiles, the number, and the identity of bovine imprinted genes that are transcribed during pre-implantation development may not be the same in embryos of other species.

Using reverse transcription polymerase chain reaction (RT-PCR) and uniparental embryos, selected imprinted genes, such as *MEST, SGCE,* and *NNAT* were found to be bi-allelically expressed in bovine *in vitro/vivo* blastocysts^12^,^41^, suggesting the late onset of monoallelic expression in the bovine. However, regardless of parental-specific allelic expression, it is a gene’s mRNA abundance and eventual translation that exerts its function. With the powerful high-throughput RNA-seq technology, we obtained profiles of mRNA abundance of all known bovine imprinted genes at multiple stages of *in vivo* development. We found that *MEST* and *GNAS* had the highest abundance in early oocytes/embryos across all species studied, suggesting conserved roles in early development. Although we were not able to distinguish the specific parental alleles from which the genes were expressed, the fact that none of the bovine imprinted genes studied to date exhibits monoallelic expression by the blastocyst stage, suggests that the gene expression we quantified was likely due to the combination of mRNA from the maternal allele in the oocytes and from both parental alleles in early embryos. The counterintuitive levels of several genes, such as *MEST* and *PLAGL1*, both paternally expressed yet highly abundant in bovine oocytes, and *PHLDA2, GNAS, MEG3, DGAT1, ASCL2* and *H19*, all maternally expressed yet barely detectable in bovine oocytes, are intriguing. These patterns suggest either the lack of genomic imprints on the maternal alleles of these genes or that these imprints are not recognized. Indeed, differential methylation at the imprinting control region of several genes including some of those characterized here, *PLAGL1* and *PEG3*, were not established during gametogenesis in non-human primates^42^. The late onset of monoallelic expression of imprinted genes in the bovine suggests that genomic imprints may also be established post-fertilization.

All 18 expressed bovine imprinted genes had developmental stage-specific dynamic patterns, which may provide insights into their specific roles in the developing embryos. For example, *MEST* and *PLAGL1* were high early in development and then decreased, indicating potential roles in oocytes, fertilization, or initial cleavage events. *PHLDA2* peaked between 2- and 4-cell stages and decreased subsequently. Although the exact role of *PHLDA2* during embryo development is unclear, when a siRNA specific to *PHLDA2* was injected into bovine zygotes a substantial increase in blastocyst development resulted^30^. These observations, together with our data, suggest that *PHLDA2* may inhibit embryonic development during the later pre-implantation period and is therefore selectively down-regulated. *CDKN1C* is another imprinted gene whose expression during bovine pre-implantation development was confirmed by functional studies. Injection of *CDKN1C-*specific siRNA into one-cell zygotes resulted in a 45% reduction in blastocyst development^30^, an observation consistent with our finding that it was up-regulated at the 16-cell stage after the initial degradation of maternal mRNA from the oocytes. The decrease in *PHLDA2* and increase in *CDKN1C* thus ensure proper blastocyst development.

A relatively large number of the bovine genes studied, 8 out of 18, either peaked or increased at the blastocyst stage. These include the maternally expressed *CDKN1C, IGF2R*, *GNAS, MEG3, DGAT1, ASCL2* and the paternally expressed *NNAT* and *NAP1L5*^43^,^44^,^45^. As we found previously^24^, a wave of increased gene expression occurs during the morula to blastocyst transition in the bovine. These eight genes may be up-regulated to prepare the bovine embryos to undergo differentiation and further development. Interestingly, these genes had very different dynamics among the different species. These patterns may reflect the differences in the speed of development and the timing of maternal-zygotic transition and differentiation among the species studied.

Eight genes were not detectable in the bovine oocytes or pre-implantation embryos while displaying relatively high levels in certain embryonic stages in other species. For example, *SNRPN, TSSC4* and *USP29,* were both imprinted and expressed in human and mouse pre-implantation embryos. Because monoallelic expression of imprinted genes is tissue- and developmental stage-specific^4^,^10^,^18^, these genes might not play a role in bovine pre-implantation development, but may be important in other species at these stages.

Additionally, our study provided information on mRNA levels relative to each other. Highly abundant mRNAs, such as those for *MEST* and *PLAGL1,* as well as lowly expressed genes, such as *H19* and *IGF2* were identified. Such information was not available from previous studies, using real time PCR where all genes were expressed as percentages of controls (set at 1 or 100%) which gives the illusion that these genes were expressed at similar levels^12^,^14^,^19^,^38^,^40^.

Lastly, we also noted differences between *in vivo* and *in vitro* produced embryos. For example, *SNRPN* and *TSSC4* were undetectable in our study, but detected in bovine *in vitro* embryos^33^. Likewise, *H19*, *IGF2* and *PEG10* were undetectable in *in vivo* embryos in pigs; however, these mRNAs had been observed in pig *in vitro* blastocysts^15^. There is evidence that *in vitro* culture and somatic cell nuclear transfer affects the establishment of *SNRPN* imprinting^14^. These differences further demonstrate that *in vitro* culture conditions can induce anomalies in genomic imprinting and imprinted gene expression and reinforce the need to use *in vivo* embryos to establish the gold standard of expression dynamics.

In summary, we provide here a reference base for the levels of imprinted genes in bovine *in vivo* produced oocytes and early embryos and contrasted these patterns with those in other species. The exact nature of genomic imprints and the timing of their establishment during early development have yet to be examined systematically. The connection between genomic imprints and actual monoallelic expression will be a major focus of our future studies.

## Methods

### Data Mining of Bovine Imprinted Genes in Pre-implantation Development

The expression profiles of bovine *in vivo* derived oocytes and pre-implantation embryos were characterized by RNA-seq and published recently^24^. Briefly two biological replicates of *in vivo* produced bovine oocytes and embryos at the 2-, 4-, 8-, early morula, late morula and blastocyst stages were subjected to RNA-seq at the depth of approximately 30 million reads per sample. High reproducibility of the biological replicates of the same developmental stage were shown by Pearson correlation coefficients and principal component analyses (PCA) in RNA-seq datasets^24^. To analyze species differences, three other RNA-seq datasets of pre-implantation development from the human, mouse and pig were downloaded from Gene Expression Omnibus (GEO) *(www.ncbi.nlm.nih.gov/geo)* under the accession numbers GSE44183^34^, and SRA076823^32^. All oocytes and embryos used in these studies were *in vivo* derived with the exception of those from humans (Supplementary Table S3). For each embryonic stage, data were normalized among the four species by transforming uniquely mapped reads to RPKM (Reads Per Kilobase of transcript per Million mapped reads)^46^. The 26 genes that have been confirmed to be imprinted in the bovine were examined in the bovine as well as in humans, mice and pigs regardless of their imprinting status in these species^9^ (Supplementary Table S4). Expression profiles of these genes were searched against all four datasets and the RPKM values of each gene from the same developmental stage were averaged and analyzed among four species. All genes with RPKM >0.1 were defined as detectable.

### Quantitative Real Time-Reverse Transcription Polymerase Chain Reaction (qRT-PCR) Analysis

Quantitative real-time PCR (qRT-PCR) was performed to validate expression of 5 selected genes (*MEST*, *PLAGL1, CDKN1C*, *PHLDA2* and *GNAS)* using bovine oocytes and embryos at the 4-cell and blastocyst stages (n = 3). Amplified RNA from individual embryos was reverse transcribed to cDNA by SuperScript III Reverse Transcriptase (Invitrogen) and amplified with specific primers (Supplementary Table S5). The qRT-PCR was performed using SYBR Green PCR Master Mix (ABI) and the ABI 7500 Fast instrument. Data were analyzed using the 7500 software version 2.0.2 provided with the instrument. All values were normalized to the internal control, *β-ACTIN*. The efficiency of each primer pair was calculated over a 3.5 log dilution range and the relative gene expression values were calculated using the 2^−△△Ct^ method. The oocytes and embryos at the 4- and blastocyst stages were pooled and used as the calibrator sample. The mean for each stage was determined and compared for an overall fold change.

## Additional Information

**How to cite this article**: Jiang, Z. *et al.* mRNA Levels of Imprinted Genes in Bovine *In Vivo* Oocytes, Embryos and Cross Species Comparisons with Humans, Mice and Pigs. *Sci. Rep.* **5**, 17898; doi: 10.1038/srep17898 (2015).

## Supplementary Material

Supplementary Table S1

Supplementary Table S2

Supplementary Table S3

Supplementary Table S4

Supplementary Table S5

## Figures and Tables

**Table 1:** Imprinting status in humans, mice and pigs for the eight genes that are imprinted, but undetectable in bovine oocytes and embryos.

Bovine imprinted genes	Human	Mouse	Pig
*APEG3*	Not imprinted	Imprinted	Not imprinted
*SNRPN*	Imprinted	Imprinted	Not imprinted
*TSSC4*	Imprinted	Imprinted	Not imprinted
*USP29*	Imprinted	Imprinted	Not imprinted
*NESP55*	Not imprinted	Not imprinted	—
*MAGEL2*	—	—	Not imprinted
*KCNQ1OT1*	—	—	Not imprinted
*MIMT1*	—	Not imprinted	Not imprinted

“ – ”: Information on imprinting status is not available.

**Table 2:** Quantitative real-time RT-PCR (qRT-PCR) results of five selected genes in oocytes, 4-cell and blastocyst stage embryos.

Gene symbol	Oocyte vs. 4-cell		Oocyte vs. Blastocyst	
	Log (Fold change) RNA-seq	Log (Fold change)*qRT-PCR	Log (Fold change) RNA-seq	Log (Fold change)*qRT-PCR
*CDKN1C*	3.0	3.6	1.8	2.5
*MEST*	1.9	2.5	9.7	9.8
*PLAGL1*	2.0	2.3	12.3	12.4
*PHLDA2*	−2.5	−2.8	−0.1	−1.4
*GNAS*	−0.6	−1.5	−4.7	−5.1

^*^Fold change is expressed as the ratios of the values of the oocyte (n = 3) divided by those of the 4-cell and blastocyst embryos (n = 3), respectively. Real time RT-PCR results substantiated the differential gene expression patterns from RNA-seq.
