# Supplementary material for: mRNA Levels of Imprinted Genes in Bovine In Vivo Oocytes, Embryos and Cross Species Comparisons with Humans, Mice and Pigs
Source: Sci Rep. 2015 Dec 7;5:17898. doi: 10.1038/srep17898 (PMC4671149; doi:10.1038/srep17898)
Supplement: Supplementary Table S3 [file srep17898-s3.doc]

|  | Cattle* | Porcine  (4-10 oocytes/embryos per group) | Mouse* | Human* |
| --- | --- | --- | --- | --- |
| Oocyte | 2 | 2 | 2 | 3 |
| Pronuclei | - | - | 3 | 3 |
| Zygotes | - | - | - | 2 |
| 2-cell | 2 | 2 | 3 | 2 |
| 4-cell | 2 | 2 | 3 | 4 |
| 8-cell | 2 | 2 | 3 | 11 |
| 16-cell | 2 | - | - | - |
| Mourla | Early morula: 2 | 2 | 3 | 3 |
|  | Compact morula: 2 |  |  |  |
| Blastocyst | 2 | 2 | - | - |

Supplementary Table S3. Summary of the numbers of biological replicates used in the four datasets of this study.

replicate

species

stage

*Single oocyte/embryo per biological replicate was used in the studies of cattle, mice and humans. All oocytes/embryos were in vivo produced with the exception of the humans.
